# Linking everyday physical activity and capacity tests using wearable and mobile technologies in older adults and cardiac cohorts: protocol for a pilot observational study

**DOI:** 10.1136/bmjopen-2025-112539

**Published:** 2026-02-12

**Authors:** Sara Caramaschi, Benjamin Maus, Carl Magnus Olsson, Daniel Smedberg, Helmuth Kristen, Millie Whitehead, Elizabeth Orchard, Dario Salvi

**Affiliations:** 1 Sustainable Digitalization Research Center, Department of Computer Science and Media Technology, Malmö University, Malmö, Skåne County, Sweden; 2 Medicon Village, Infonomy, Lund, Skåne County, Sweden; 3 Cardiac Physiology, Oxford University Hospitals NHS Foundation Trust, Oxford, UK; 4 Oxford University Hospitals NHS Foundation Trust, Oxford, England, UK

**Keywords:** Telemedicine, Congenital heart disease, Digital Technology, Exercise Test, Frail Elderly, Physical Fitness

## Abstract

**Abstract:**

**Introduction:**

This study investigates the potential of digital health technologies (DHTs), such as wearable devices and smartphones, to complement traditional submaximal functional capacity tests, such as the 6 min walk test (6MWT) and the timed up and go test (TUG). While these traditional tests are widely used due to their simplicity and relevance to daily living activities, they have limitations, including infrequent administration and the need for clinical observation. DHT offers continuous, real-world monitoring, which may accurately reflect patients’ health status and effectively inform clinical decisions. However, there is a need to establish the validity of the data and metrics computed through DHT and understand patient perspectives on using such technology.

**Methods and analysis:**

This is an observational pilot study (Synergy Digital Health study) that aims at linking wearable data with traditional test outcomes and assessing participants’ acceptance and usage of such DHT. A cohort of 30 cardiovascular patients from Oxford University Hospitals, UK, and 30 community-dwelling elderly people from social centres in Helsingborg, Sweden, will use wearable devices for 2 months in free-living conditions, they will fill out technology acceptance questionnaires (AQs), have baseline assessments and perform physical tests such as the 6MWT and TUG using the Mobistudy smartphone app. Subgroups will participate in codesign workshops to identify experience-based design recommendations for the technology. Quantitative and qualitative methods will be adopted to analyse the collected data.

**Ethics and dissemination:**

The study protocol received ethical approval in Sweden from the Etikprövningsmyndigheten (2024-04886-01) and in the UK from the National Health Service (NHS) Research Ethics Committees (Iras project ID: 340870), in accordance with local regulations. All participants are asked for written informed consent. The results of the study will be shared via scientific journals and conferences.

**STRENGTHS AND LIMITATIONS OF THIS STUDY**

- This study makes use of quantitative and qualitative methods to investigate the use of consumer technologies for health monitoring.
- The study evaluates the construct validity of wearable data collected in free-living conditions by identifying associations with standard physical function and capacity tests.
- This research considers machine learning methods, time series and statistical approaches while including data reliability assessments, which play a crucial role for data collected in free-living conditions.
- The study considers participants’ perspectives on the technologies through interviews and workshops.
- This study presents some risks reflected in the differences between the two cohorts (older adults and cardiac patients), in the use of technology that may present defects and in the possible limited variability of participants’ mobility.

## Introduction

 Many health issues, such as pulmonary, neurological and heart diseases, can limit physical mobility and worsen limb functioning over time. Clinicians use submaximal function and capacity tests such as the 6 min walk test (6MWT) and the timed up and go test (TUG) to monitor patients’ mobility status, disease progression^1 2^ and overall health.^3^ The 6MWT measures the distance walked over a 6-minute window. This test is usually carried out in a hospital corridor, and it is typically employed to measure exercise capacity, the maximum level of physical exertion that one can sustain.^4^ The TUG test measures the time required by the patient to stand up from a chair, walk three metres, turn around, walk back and sit down again. It is commonly used to measure lower extremity function, mobility and fall risk.^5^ The 6MWT and TUG are widely used in clinical practice because they are easy to administer, safe and representative of daily living activity. This is especially crucial for patients with or at risk of limited mobility who significantly struggle to perform the gold standard maximal test, the cardiopulmonary exercise test.^6^ In these cases, the submaximal 6MWT and TUG are suitable for administration at follow-up appointments to objectively assess changes in symptoms, physical function and capacity and overall health status. These tools are commonly used in clinical studies, for example, with elderly people, where the 6MWT was found to indicate overall health,^7^ and in cardiac patients, such as those who underwent the Fontan procedure.^8^

The tests, particularly the 6MWT given its focus on endurance, may benefit patients who undergo the Fontan-Kreutzer procedure, a surgery performed on children born with congenital heart disease who lack two functional ventricles and an effective parallel blood flow circuit.^8 9^ These patients require low pulmonary vascular resistance and high systemic venous pressures to drive blood through the pulmonary vasculature, which leads to reduced cardiorespiratory functional capacity and altered skeletal muscle function, ultimately limiting their exercise capacity.^10^ For them, the 6MWT provides a simple, submaximum test for regular monitoring.^11 12^ Despite its validated representation of physical function and link to capacity,^13^ the TUG is less commonly used among Fontan populations. It is therefore relevant to investigate its association and complementarity in comparison to the 6MWT. The TUG is instead prevalent when assessing older adults, for example, to predict or investigate risk of falling, muscle strength and gait.^3 14^ The 6MWT is also used for a general elderly population, given its association with lower limb functioning and balance across a wide body of scientific literature.^15 16^ Both tests are highly indicative of general health status in this population, independently of the nature of any health condition and associated with the risk of falls.^17^ Notwithstanding the simplicity of these tests, they present some relevant shortcomings: they are at best performed once or twice per year, they require the patient to travel to the clinic, and they incur costs for hospitals given the clinical staff needed to observe the tests.

In this context, digital health technologies (DHTs),^18 19^ such as wearable devices (eg, fitness trackers, smartwatches) and smartphones, can continuously track physical activity in everyday life, that is, the ‘physical performance’ defined by the International Classification of Functioning, Disability and Health.^20^ Measuring physical performance can be used to complement—or even replace—standard physical tests for function and capacity.^21^ Given that wearables measure activity longitudinally and under different conditions, the data they produce can reflect users’ health status, complementing infrequent in-clinic tests, and therefore support clinicians as they assess patients’ health, adjust medications and recommend interventions. A summarised view of everyday physical activity data has the potential to provide a more comprehensive representation of health fluctuations over time, supporting self-monitoring and health engagement.^22 23^ Despite the high potential of DHTs, there is a lack of consensus and standard procedures for processing and using continuous data collected from these devices from a clinical perspective.^24^ Their adoption in healthcare requires robust validation across diverse populations and environments, and against standardised clinical endpoints, such as physical tests.

The primary aim of this study is thus to assess if a link exists between movement quantified through wearable and mobile devices and standard clinical tests such as the 6MWT and TUG. Identifying such a link will help include data from DHTs within clinical workflows.

In addition to the potential benefits of incorporating DHT into clinical practices, it is crucial to consider the patient’s perspective as an end-user of this technology. Factors like digital inclusion and literacy,^25^ privacy and trust,^26^ as well as technology acceptance and adoption^27^ pose significant challenges when integrating these devices into the daily lives of patients, who may not yet be familiar with wearables. The continuous sharing of data with healthcare professionals is also not universally accepted, and the literature lacks sufficient characterisation of how patients perceive using this technology for monitoring their movement and daily physical activity behaviour.^28^ This focus is particularly relevant for adults with Fontan circulation as a rare and complex congenital heart condition. Despite the increasing availability of digital health tools, little is known about how Fontan patients use technology as a self-management tool in their everyday lives.

As a secondary aim, we evaluate participants’ acceptance of the remote monitoring technology used in the study, to build on their needs and experiences to inform the design of such technology. This includes assessing participants’ attitude towards the technology before, during and after the study, focusing on mediators such as perceived health threat, perceived usefulness and perceived ease of use. Understanding these aspects in combination with an explorative workshop format will provide insights into a user-centred design of such technology, which is crucial for its effective implementation and integration into routine patient care.

The following are the research questions (RQs) that motivate our study:

*RQ 1*. What is the relationship between physical activity measurements collected in a free-living environment from DHT and the outcomes of standard physical function tests such as the 6MWT and the TUG?

*RQ 2*. What is the reliability of tests performed using mobile phones?

*RQ 3*. To what extent do the participants accept and use the technology examined?

*RQ 4*. What design considerations emerge from cardiac patients’ and community-dwelling older adults’ experiences with DHT for monitoring function and capacity?

*RQ 5*. Are the results of the Timed Up and Go test associated to the results of the 6MWT?

## Methods and analysis

This is an observational pilot study (Synergy Digital Health study) that aims to investigate data collected in free-living conditions and standard physical tests in two specific cohorts. It investigates and validates recruitment, data collection and measurement protocols, informing the feasibility of a larger-scale study. This research involves participants from two different cohorts, varying in age range and health conditions. Despite the differences between the two cohorts, both groups would benefit from monitoring their physical function and capacity using wearable devices and smartphones. Participants will perform two conventional physical tests, 6MWT and TUG, answer questionnaires and wear wearable devices for 2 months.

### Participants

One cohort consists of 30 Fontan procedure patients under treatment at the Oxford University Hospitals (OUH) Trust, Oxford, United Kingdom. The second cohort corresponds to 30 community-dwelling elderly persons recruited at meeting points in the city of Helsingborg (HBG), Sweden.

Potential participants from previous studies in relation to the smartphone-based 6MWT provided their feedback and were involved in the design of the current study.

Table 1 reports the inclusion and exclusion criteria for the two cohorts.

**Table 1:** Inclusion and exclusion criteria for the Synergy Digital Health study participants

Oxford University Hospitals (OUH)		Helsingborg (HBG)	
Inclusion criteria	Exclusion criteria	Inclusion criteria	Exclusion criteria
Age 18 years or above. The participant can walk at least one kilometre safely. The participant uses a smartphone. Fontan procedure patient under the care of the Congenital Heart Disease clinic at OUH. Understanding of verbal and written English.	Long-term oxygen therapy. Cognitive impairments. Cannot use a smartphone. Pregnancy. Not able to complete a 6 min walk test (6MWT) or a timed up and go test (TUG) test.	Aged 65 years or above. The participant can walk at least 1 kilometre safely. The participant uses a smartphone. The participant lives in HBG and/or regularly visits a meeting point for seniors in HBG. Understanding of verbal and written Swedish.	Long-term oxygen therapy. Cognitive impairments. Cannot use a smartphone. Not able to reach meeting points in HBG. Not able to complete a 6MWT or a TUG test.

HBG, Helsingborg; 6MWT, 6 min walk test; OUH, Oxford University Hospitals; TUG, timed up and go test.

### Sample size

Literature shows that the correlation between gait-related features obtained passively and conventional 6MWD ranges from 0.21 (p<0.01),^29^ 0.48^30^ and 0.68 (p<0.01).^31^ We set our expected correlation coefficient to 0.4, from which we determined that a minimum of 47 participants is required to reject the null hypothesis (no correlation). For this estimate, the threshold probability for rejecting the null hypothesis (type I error, alpha) is set at 0.05. In contrast, the probability of failing to reject the null hypothesis under the alternative hypothesis (type II error, beta) is set to 0.2. Our sample size of 60 should thus suffice even in the presence of dropouts. To guarantee variability in the data and increase overall external validity, participants will be selected to maximise variation in mobility, from relatively healthy to more impaired individuals. Age and sex will also be considered to avoid overrepresentation within either of those variables.

### Technology

The so-called DHTs used in this study are participants’ smartphones, the Xiaomi Mi Band 3 (Xiaomi Global, https://www.mi.com/global/) and the Snubblometer (Infonomy AB, https://infonomy.com/). In addition, during the first baseline visit, the medical-grade device G-Walk (BTS Bioengineering, https://www.btsbioengineering.com/) is used as an additional reference to the standard clinician assessment and to the reference collected via a trundle wheel. This device selection includes a medical-grade device for use in clinic, and consumer off-the-shelf devices for home monitoring. This allows the alignment of robust and validated devices, inexpensive consumer devices and reference collected through standard ways from researchers and clinicians (using a stopwatch for the TUG or measuring the covered walked distance considering a known path for the 6MWT).

Participants are asked to install the Mobistudy^32^ app on their smartphones. This app is developed by Malmö University and includes features such as electronic consent, forms and surveys, data synchronisation with the Xiaomi Mi Band 3 fitness tracker, guided 6MWT and TUG tests with the collection of sensor data for the computation of the outcomes (6MWT distance and TUG time). The wrist-worn Xiaomi Mi Band 3 device collects information about step counting, activity, sleep and heart rate every minute. The device can synchronise its data with the Mobistudy app via Bluetooth without the need for additional software. The Snubblometer is a leg-worn device that collects inertial data (accelerometer and gyroscope) and, through internal algorithms, computes multiple metrics related to the user’s gait such as step length, step velocity, sit-to-stand velocity and body position (lying on left, right or centre and standing). The G-Walk, used only during the baseline assessment, is a medical-grade device for gait analysis. It is placed on the lower back and collects gait-related information through inertial sensors (triaxial accelerometer, triaxial gyroscope, triaxial magnetometer). The device provides relevant metrics during the 6MWT and TUG, including duration, speed and distance. These will be used as a reference for validating algorithms developed for the Mobistudy app.

Except the G-Walk, none of the other devices is intended for medical use. This study does not aim at validating a medical device, but at assessing the potential of using non-medical consumer technologies within healthcare.

### Study design

**Figure 1:**
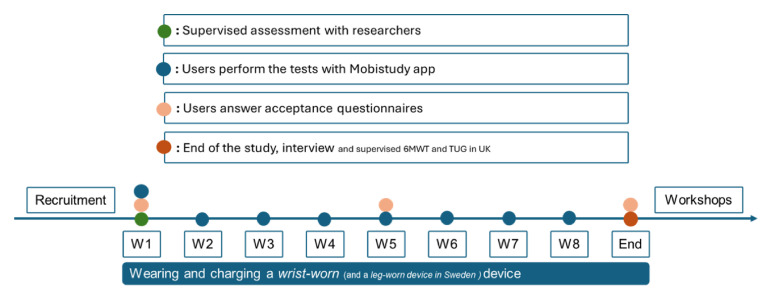
Temporal summary of the study including the procedures that participants will undergo. 6MWT, 6 min walk test; TUG, timed up and go test.

Figure 1 shows a temporal summary of the study design. Following are the steps that will characterise participants’ enrolment in the study:

1. Recruitment: The recruitment in HBG of elderly participants will happen through public events held by the City Council. The recruitment at OUH of Fontan circulation patients will happen through regularly scheduled visits at the hospital.
2. First visit: During the first visit, participants are introduced to the project and asked to complete the consent form. If the consent form is signed, the participant is guided through the set-up of the smartphone app and the wearable devices. After setup, participants will perform a supervised 6MWT and TUG test using both their smartphones with the Mobistudy app, and the G-Walk and the trundle wheel for reference measurements. After the completion of the tests, they will be asked to answer a first AQ (Q1) using the app. The time duration of the first visit is estimated to be around 1 hour.
3. Passive measurements: Once the first visit is completed, participants are asked to continuously wear the Xiaomi Mi Band 3 for 2 months during their regular life and to synchronise the data collected by the fitness tracker with the Mobistudy app. Participants from HBG will also wear the Snubblometer on one thigh, the data is synchronised when the device is put to charge, through an Internet-connected base station. The Snubblometer will not be used for the OUH cohort given that the device nature is targeted at an elderly population, and it may not be as useful for a younger group.
4. Active measurements: Once a week, the Mobistudy app will remind participants to perform physical tests with the support of their own smartphone. The cohort in HBG will perform weekly 6MWT and the TUG tests, while the cohort in UK will perform weekly 6MWT. This is in order to reduce the burden on Fontan procedure patients that can focus on the performance of the 6MWT at home. The 6MWT is performed outdoors, where the position of the phone can be tracked, and the total distance can be measured. The TUG is performed at home, with a walkway of 3 metres and a chair. The tests can be executed in succession, taking about 30 min of time between preparation and execution.
5. Mid study: After 1 month from the start of the study, participants will be asked to answer a second AQ (Q2) similar to the first one, using the smartphone app.
6. Final visit: At the end of the study, participants will return the devices and answer a final AQ (Q3). About 50% of the participants will be interviewed by a researcher, where notes and audio recordings will be taken if they consent. At the OUH site, participants will additionally perform a final supervised set of TUG and 6MWT tests, with the same device set up as the test done in the initial visit.
7. Feedback: After completion of the study, participants will receive a summary report of the data collected during the study, which they can use to reflect on their physical activity and during discussions with their carers or social services.
8. Codesign workshop: A subset of participants, 10 per site, will be invited to join two experience-based codesign workshops aimed at gaining insights and feedback regarding the use of the technology and ideating future design features.

Data collection started in Q2 2025 and is expected to end in Q2 2026. Analysis of the data will be completed before Q4 2028.

### Data collection and analysis

The study, being data-intensive, involves collecting various data types. Provided that most data samples will be collected under unsupervised conditions, data quality and reliability will be investigated across all data sources as a cornerstone of the overall analysis. In particular, data collected from the smartphone during physical tests will be subject to quality assurance checks. These include ensuring that the duration of the test does not exceed reasonable thresholds, and that the sampling frequency of inertial and location data are sound (50 Hz for inertial and 1 Hz for location). The location data of the 6MWT will undergo filtering according to the signal quality.^33^ At the same time, for the TUG test, we will observe the quality of the smartphone orientation signal which is used to identify turn-around phases of the test. Regarding data collected through the wrist-worn device, we define requirements for wearing time duration at the hour-level (at least 45 min of available data per hour), at daily-level (at least 12 hours per day), at weekly-level (at least 3 days per week) and for the whole monitoring period (at least 2 weeks out of 8).^34^,^36^ In addition to duration-based assessment, we impose physiological boundaries on step count and heart rate values. Through these approaches, we will obtain reliable data to progress into the study’s RQs.

To address RQ1, data from the previously described DHT will undergo statistical analysis, signal processing and machine learning. The overarching goal is to identify aggregated indicators from time series data (step count, body position, heart rate, activity level, activity type) that correlates with the 6MWT (walked distance, 6MWD) and TUG test (duration, TUGD). This implies two major steps. First, statistical approaches will be used to investigate the association between aggregated indicators and the test results. In particular, we will consider equivalence testing,^37^ Pearson and Spearman correlation coefficients with significance thresholds at 0.05 and 0.001. In the statistical analysis, we will consider confounding factors such as age, sex, comorbidities and the use of walking aids through generalised linear mixed models.

Participants will perform multiple 6MWT and TUG tests over the 2-month study period. Each test will be associated with aggregates of the time series data within a week from the date of the test. These tests have excellent reproducibility and reliability within short time windows up to 1 year.^38 39^ Thus, within the 2 months of monitoring, there are no expectations of seeing substantial changes in participants’ function and capacity, as reflected in the results of the physical tests, unless major events occur within the time window.

A second step, a machine learning approach will be set up to infer the test results (6MWD and TUGD) from multiple aggregated indicators. The methods that we will adopt in this context include feature engineering techniques, time-series analysis and machine-learning methods from baseline logistic regression, as also proposed by Rens *et al*^40^ to more complex architectures, including neural networks ranging from spatial data filtering (convolutional neural networks) to sequential-based architectures (recurrent and long-short term memory neural networks).^41^ The literature lacks investigation into deep learning techniques for estimating 6MWD.

The construct validity of the developed methods for inferring physical test outcomes will be assessed to answer RQ1. See table 2 for a summary of devices, data and schedule related to RQ1.

**Table 2:** Data collection employed to address research question RQ1

Device	Collected data	Schedule
Phone (through Mobistudy app)	6MWT distance (6MWD), timed up and go test duration (TUGD), inertial sensors raw data (accelerometry, rotation rate)	Once a week, 8 weeks
Xiaomi Mi Band 3	Step count, heart rate, activity level, activity type (resting, walking, running, sleeping)	Worn every day as much time as possible for 2 months
Snubblometer	Step count, body position (standing up, lying down)	Worn every day as much time as possible for 2 months

#### Outcome

Correlation metrics between wearable devices collected data and reference tests outcome (6MWD, TUGD).

To address RQ2 (table 3), the results of 6MWT and the TUG tests produced by the phone will be compared with the reference values from either the G-Walk or trundle wheel. The statistical investigation will assess the reliability and validity of the measurements. It will include intraclass correlation coefficient analysis, and standard statistics such as mean absolute error, standard deviation of the error and Bland-Altman limits of agreement. In addition, to investigate RQ4, we will statistically compare the results of the supervised 6MWT with those of the TUG to examine the relationship between these two tests. This is particularly relevant for the Fontan procedure population, as the TUG is easier to perform and it is a validated function capacity indicator.^13^

**Table 3:** Data collection employed to address research question RQ2 and RQ4

Device	Collected data	Schedule
G-Walk	6MWT distance (6MWD), timed up and go test duration (TUGD), inertial sensors raw data (accelerometry, rotation rate)	At baseline, in clinic
Phone (through Mobistudy app)	6MWD, TUGD, inertial sensors raw data (accelerometry, rotation rate)	Once a week, 8 weeks

#### Outcome

Bland-Altman limits of agreement between phone 6MWD and G-Walk 6MWD, and between phone TUGD and G-Walk TUGD.

To address RQ3, we adopted Nadal’s Technology Acceptance Lifecycle (TAL)^42^ as the foundational framework, thus assessing the user acceptance of the technology. The TAL framework is based on a comprehensive and well-cited review of technology acceptance models within the mobile health (mHealth) domain. It aims to measure technology acceptance across three different stages of a user journey, which Nadal defines as (1) pre-use acceptability, (2) initial use acceptance and (3) sustained use acceptance. Unlike conventional approaches reliant on singular questionnaires, the TAL accounts for the evolution of user acceptance over time. More specifically, the participant completes an AQ before, during and after interacting with the solution. In combination with semistructured interviews at the end of the study, this approach provides stronger and more nuanced validation compared with traditional questionnaires.

The TAL approach builds on the Health Information Technology Acceptance Model,^43^ which had been previously identified as one extension of technology acceptance models that cover most acceptance factors relevant to DHT.^44^ We further adapted the AQ from Nadal *et al*^44^ to the context of the project and plan to use it at three different moments before, during and after the 8-week study to assess pre-use acceptability (Q1), initial use acceptance (Q2) and sustained use acceptance (Q3). See online supplemental tables S1–S3 for questionnaire details.

The semistructured interviews at the end of the study will be used to gather a deeper understanding of factors influencing the use of the technology examined. The interview questions focus on mediators that have not been explored in the AQs (see online supplemental material). Finally, experience-based codesign workshops^45^ will complement this methodological approach to address RQ5 in conjunction with the interviews. The workshops will engage participants in sharing and reflecting on their everyday experiences, generating design considerations for how the technology could better support their daily lives. While codesign is often applied early in the design process,^46^ involving users after they have interacted with a DHT offers a valuable and less-explored perspective, particularly when working with older adults.^45^ The outline of the workshop can also be found in online supplemental material.

The collected data will be analysed with a mix of quantitative methods, such as descriptive statistics of scale-type questions, and qualitative methods, such as thematic content analysis. Table 4 specifies the set up to collect the data for answering RQ3.

**Table 4:** Data collection employed to address research question RQ3

Device	Collected data	Schedule
Phone (through Mobistudy app)	Acceptance questionnaires	At baseline, after 2 weeks, after 8 weeks
	Interviews	At the end of the study
	Experience-based codesign workshop	At the end of the study

#### Outcome

Average rating of the answers provided to the Likert-scale questions in AQs. Qualitative analysis of interviews and workshops.

### Data management

Data collected by researchers (demographics, audio recording, notes), as well as the software needed to extract information from the G-Walk, will be collected on computers used by one researcher at Malmö University (HBG) and one at Oxford University NHS Trust (OUH) for the participants of the respective sites. These data are personally identifiable.

Data collected by the Mobistudy app will be stored on servers managed by Malmö University, also located at the University premises, using the institution’s strict security policies. The server can only be accessed by a researcher from the University and its IT department. Given that Mobistudy requires users to register (for reasons due to unavoidable password recovery), these data are personally identifiable.

Data collected by the Snubblometer is sent to servers managed by Infonomy AB, Sweden. The data will be downloaded by employees of the company for analysis and technical inspection, within the scope of the RQs of this study. These data will be pseudonymised with participants codes. The code/participant table will be kept by Malmö University.

After the data has been analysed by the research team (no later than 2028), the data will be deleted from the researchers’ computers and servers and archived. Archival rules in the UK and Sweden imply that the data collected at each site, independently, will be archived at the two institutions acting as sponsors: Malmö University for Sweden and OUH for the UK. The modalities and duration of the archival will follow local policies and legislation.

At the end of the data analysis, data collected by Malmö University will be anonymised and shared with the research community through specialised research databases (eg, Physionet, Dryad), following the FAIR principles (Findable, Accessible, Interoperable, and Reusable). The anonymisation process will remove any information that could lead to identification, including:

- All personal details such as names, email addresses or phone numbers will be removed.
- Timestamps will be made relative to the start of the data collection for each subject.
- Ages will be only stored in ranges.
- Geolocation data collected during the app-based 6MWT will be moved to a random location, while preserving the shape of the walked path.
- Voice recordings will be removed, and transcriptions will not include any personally identifiable information.

This is in accordance with existing guidelines.^47^

### Patient and public involvement

For the UK cohort, patients with a Fontan circulation have been involved in designing the study. For the Swedish cohort, people from the municipality responsible for care and wellbeing of older adults were involved in the study design. At the end of the study, each participant will be provided with a summary report of the data collected during the monitoring period. The summary can be discussed with healthcare professionals who are in contact with the participant.

## Ethics and dissemination

There are several challenges with using this type of technology in healthcare, including digital inclusion, data availability, privacy, third-party involvement, data quality and potential adverse effects.^35^

Certain groups may be excluded due to costs or difficulty using the technology, though usability and affordability are improving (eg, modern Mi Band trackers cost under €50). Privacy concerns arise when tech manufacturers collect health data, often in countries with weaker data protection. The Mobistudy app addresses this by extracting data directly from the MiBand, avoiding third-party apps.

Data quality is another concern, as consumer technologies are seldom used clinically due to uncertain accuracy. Reliable data is crucial for medical decisions, yet wearable devices manufacturers rarely disclose measurement accuracy. Research such as this is essential for understanding the potential and limitations of these technologies, with improvements expected in accuracy and reliability expected over time.

The study protocol obtained ethical approval in Sweden from the Swedish Ethical Review Authority: EPM 2024-04886-01 and from the NHS Health Research Authority, United Kingdom: IRAS project ID 340870. All participants are required to sign a written informed consent prior to the start of the study. We will analyse the collected data and write the results of our analysis in papers and reports to be published in scientific journals or to be presented at scientific conferences. No personal information will be disclosed in those publications. The study results will be reported following the Strengthening the Reporting of Observational Studies in Epidemiology (STROBE) recommendations for observational studies.

## Discussion

This study investigates how data collected in participants’ everyday environments, using wearable devices and smartphones, can reflect physical capacity and function when compared with standard physical tests such as the 6MWT and the TUG. The findings aim to deepen the understanding of how remotely collected data relate to standard clinical assessments, potentially enabling more continuous and cost-effective monitoring of patients’ health. The study also explores how such technologies can be meaningfully integrated into daily life by building on participants’ concrete experiences. This study comes with its own limitations. First, we use a specific wearable device that is no longer commercially available, which may limit the applicability and generalisability of our findings to other currently available devices. Additionally, the Snubblometer will be used only at a single study location (HBG), thus limiting the data available for the other location. This is due to the fact that the device is tailored particularly for the elderly population and is therefore not of relevance for the OUH cohort. The recruitment process also poses limitations, as, in one study location (HBG), older adults are enrolled through an open-call initiative rather than through clinics. It is possible that older adults willing to participate will enjoy relatively good health, potentially leading to little variation in mobility and affecting comparability across groups. Finally, the qualitative data collected in this study is based on subjective, attitudinal reflections. A longitudinal approach could provide more stability and depth in these reflections in comparison to the current approach.

Despite its limitations, this study still has the potential to reveal insights and relationships between collected data and physical capacity in cohort contexts such as one of Fontan procedure patients in OUH and the elderly in HBG. It explores the potential of wearable devices and smartphones to complement or replace traditional clinical tests, offering continuous, real-world monitoring that could provide a more comprehensive assessment of patients’ health. By considering the perspectives of patients as end-users of wearable technology, it addresses important factors such as everyday needs, digital literacy, privacy concerns and technology acceptance, which are crucial for successful implementation in clinical practice.

### Data availability statement

Collected data will be anonymised and shared with the research community through specialised research databases (eg, Physionet, Dryad), following FAIR principles.

## Supplementary material

10.1136/bmjopen-2025-112539 online supplemental file 1
