# Supplementary material for: Linking everyday physical activity and capacity tests using wearable and mobile technologies in older adults and cardiac cohorts: protocol for a pilot observational study
Source: BMJ Open. 2026 Feb 12;16(2):e112539. doi: 10.1136/bmjopen-2025-112539 (PMC12911791; doi:10.1136/bmjopen-2025-112539)
Supplement: online supplemental file 1 [file bmjopen-16-2-s001.docx]

Supplemental Material

**Acceptance questionnaire (AQ) and open-ended questions**

**Table S1: AQ at Day 0 (T1)**

| Mediator | Device | Item codes | Measurement items |
| --- | --- | --- | --- |
| Perceived Threat | N/A | PT1 | I am strongly concerned about my physical health. |
|  |  | PT2 | I would make efforts to manage my physical health. |
| Perceived Usefulness | wrist-worn device | PUW1 | I think that measuring my physical activity with the wrist-worn device will likely help in managing my physical health. |
|  |  | PUW2 | I think that measuring my heart activity with the wrist-worn device will likely help in managing my physical health. |
|  | only OUH | PUW3 | I think that sharing data collected by the wrist-worn device with my clinician will likely be helpful in managing my physical health. |
|  | leg-worn device (only HBG) | PUL1 | I think that measuring aspects related to my walking with the leg-worn device will likely help in managing my physical health. |
|  |  | PUL2 | I think that measuring aspects related to my body position with the leg-worn device will likely help in managing my physical health. |
|  |  | PUL3 | I think that sharing data collected by the leg-worn device with my clinician will likely be helpful in managing my physical health. |
|  | app | PUA1 | I think that doing a 6-minute walk test with the Mobistudy app will likely help in managing my physical health. |
|  |  | PUA2 | I think that doing a timed up and go test with the Mobistudy app will likely help in managing my physical health. |
| Perceived Ease of Use | wrist-worn device | PEOUW1 | I think that the wrist-worn device will be easy to put on, wear and charge. |
|  | leg-worn device (only HBG) | PEOUL1 | I think that the leg-worn device will be easy to put on, wear and charge. |
|  | app | PEOUA1 | I think that it will be easy to do a 6-minute walk test with the Mobistudy app. |
|  |  | PEOUA2 | I think that it will be easy to do a timed up and go test with the Mobistudy app. |
|  |  | PEOUA3 | I think that it will be easy to connect the Mobistudy app with the wrist-worn device. |
| Attitude | wearables | AW1 | I am positive towards recording my physical activity data with wearable devices. |
|  |  | AW2 | I am positive towards recording my heart activity data with wearable devices. |
|  |  | AW3 | I am positive towards sharing my physical activity data for research purposes. |
|  |  | AW4 | I am positive towards sharing my heart activity data for research purposes. |
|  |  | AW5 | I want to have access to and review my own data. |
|  |  | AW6 | I want my clinician to have access to and review my data. |
|  |  | AW7 | I want my family (or other close contact) to have access to and review my data. |
|  | HBG only | AL1 | I am positive towards recording data related to walking using wearable devices. |
|  |  | AL2 | I am positive towards recording my date related to my body position using wearable devices. |
|  |  | AL3 | I am positive towards sharing data related to my walking  for research purposes. |
|  |  | AL4 | I am positive towards sharing data related to my body position data for research purposes. |
| Behavioural Intention | wrist-worn device | BIW1 | I intend to use the wrist-worn device until completion of the study. |
|  | leg-worn device (HBG only) | BIL1 | I intend to use the leg-worn device until completion of the study. |
|  | app | BIA1 | I intend to use the app until completion of the study. |
| Usage Behaviour | N/A | | |

**Table S2: AQ at Day 30 (T2)**

| Mediator | Device | Item codes | Measurement items |
| --- | --- | --- | --- |
| Perceived Threat | N/A | PT1 | I am strongly concerned about my physical health. |
|  |  | PT2 | I would make efforts to manage my physical health. |
| Perceived Usefulness | wrist-worn device | PUW1 | I think that measuring my physical activity with the wrist-worn device is likely helpful in managing my physical health. |
|  |  | PUW2 | I think that measuring my heart activity with the wrist-worn device is likely helpful in managing my physical health. |
|  | only OUH | PUW3 | I think that sharing data collected by the wrist-worn device with my clinician is likely helpful in managing my physical health. |
|  | leg-worn device (only HBG) | PUL1 | I think that measuring aspects related to my walking with the leg-worn device is likely helpful in managing my physical health. |
|  |  | PUL2 | I think that measuring aspects related to my body position with the leg-worn device is likely helpful in managing my physical health. |
|  |  | PUL3 | I think that sharing data collected by the leg-worn device with my clinician is likely helpful in managing my physical health. |
|  | app | PUA1 | I think that doing a 6-minute walk test with the Mobistudy app is likely helpful to manage my physical health. |
|  |  | PUA2 | I think that doing a timed up and go test with the Mobistudy app is likely helpful to manage my physical health. |
| Perceived Ease of Use | wrist-worn device | PEOUW1 | I think that the wrist-worn device is easy to put on, wear and charge. |
|  | leg-worn device (only HBG) | PEOUL1 | I think that the leg-worn device is easy to put on, wear and charge. |
|  | app | PEOUA1 | I think that it is easy to do a 6-minute walk test with the Mobistudy app. |
|  |  | PEOUA2 | I think that it is easy to do a timed up and go test with the Mobistudy app. |
|  |  | PEOUA3 | I think that it is easy  to connect the Mobistudy app with the wrist-worn device. |
| Attitude | wearables | AW1 | I am positive towards recording my physical activity data with wearable devices. |
|  |  | AW2 | I am positive towards recording my heart activity data with wearable devices. |
|  |  | AW3 | I am positive towards sharing my physical activity data for research purposes. |
|  |  | AW4 | I am positive towards sharing my heart activity data for research purposes. |
|  |  | AW5 | I want to have access to and review my own data. |
|  |  | AW6 | I want my clinician to have access to and review my data. |
|  |  | AW7 | I want my family (or other close contact) to have access to and review my data. |
|  | HBG only | AL1 | I am positive towards recording data related to walking using wearable devices. |
|  |  | AL2 | I am positive towards recording my date related to my body position using wearable devices. |
|  |  | AL3 | I am positive towards sharing data related to my walking  for research purposes. |
|  |  | AL4 | I am positive towards sharing data related to my body position data for research purposes. |
| Behavioural Intention | wrist-worn device | BIW1 | I intend to use the wrist-worn device until completion of the study. |
|  | leg-worn device (only HBG) | BIL1 | I intend to use the leg-worn device until completion of the study. |
|  | app | BIA1 | I intend to use the app until completion of the study. |
| Usage Behaviour | wrist-worn device | UBW1 | How would you describe your use of the wrist-worn device? |
|  |  | UBW2 | What difficulties (if any) did you experience when using the wrist-worn device? |
|  | leg-worn device (only HBG) | UBL1 | How would you describe your use of the leg-worn device? |
|  |  | UBW2 | What difficulties (if any) did you experience when using the leg-worn device? |
|  | app | UBA1 | How would you describe your use of the Mobistudy app? |
|  |  | UBA2 | What difficulties (if any) did you experience when using the Mobistudy app? |

**Table S3: AQ at Day 60 (T3)**

DS1: Have you received and looked at your data summary?

DS2: I think that it was easy to understand the data in the summary.

DS3: What would you have wished to see on the data summary (if anything)?

DS4: What was hard to understand for you on the data summary (if anything)?

DS5: What was particularly interesting (if anything) for you regarding the data summary?

| Mediator | Device | Item codes | Measurement items |
| --- | --- | --- | --- |
| Perceived Threat | N/A | PT1 | I am strongly concerned about my physical health. |
|  |  | PT2 | I would make efforts to manage my physical health. |
| Perceived Usefulness | wrist-worn device | PUW1 | I think that measuring my physical activity with the wrist-worn device is likely helpful in managing my physical health. |
|  |  | PUW2 | I think that measuring my heart activity with the wrist-worn device is likely helpful in managing my physical health. |
|  | only OUH | PUW3 | I think that sharing data collected by the wrist-worn device with my clinician is likely helpful in managing my physical health. |
|  | leg-worn device (only HBG) | PUL1 | I think that measuring aspects related to my walking with the leg-worn device is likely helpful in managing my physical health. |
|  |  | PUL2 | I think that measuring aspects related to my body position with the leg-worn device is likely helpful in managing my physical health. |
|  |  | PUL3 | I think that sharing data collected by the leg-worn device with my clinician is likely helpful in managing my physical health. |
|  | app | PUA1 | I think that doing a 6-minute walk test with the Mobistudy app is likely helpful to manage my physical health. |
|  |  | PUA2 | I think that doing a timed up and go test with the Mobistudy app is likely helpful to manage my physical health. |
| Perceived Ease of Use | wrist-worn device | PEOUW1 | I think that the wrist-worn device was easy to put on, wear and charge. |
|  | leg-worn device (only HBG) | PEOUL1 | I think that the leg-worn device was easy to put on, wear and charge. |
|  | app | PEOUA1 | I think that it was easy to do a 6-minute walk test with the Mobistudy app. |
|  |  | PEOUA2 | I think that it was easy to do a timed up and go test with the Mobistudy app. |
|  |  | PEOUA3 | I think that it was easy to connect the Mobistudy app with the wrist-worn device. |
| Attitude | wearables | AW1 | I am positive towards recording my physical activity data with wearable devices. |
|  |  | AW2 | I am positive towards recording my heart activity data with wearable devices. |
|  |  | AW3 | I am positive towards sharing my physical activity data for research purposes. |
|  |  | AW4 | I am positive towards sharing my heart activity data for research purposes. |
|  |  | AW5 | I want to have access to and review my own data. |
|  |  | AW6 | I want my clinician to have access to and review my data. |
|  |  | AW7 | I want my family (or other close contact) to have access to and review my data. |
|  | HBG only | AL1 | I am positive towards recording data related to walking using wearable devices. |
|  |  | AL2 | I am positive towards recording my date related to my body position using wearable devices. |
|  |  | AL3 | I am positive towards sharing data related to my walking  for research purposes. |
|  |  | AL4 | I am positive towards sharing data related to my body position data for research purposes. |
| Behavioural Intention | wrist-worn device | BIW1 | I would use the wrist-worn device in the future to measure my physical activity or my heart activity. |
|  | leg-worn device (only HBG) | BIL1 | I would use the leg-worn device in the future to measure aspects of my walking or body position. |
|  | app | BIA1 | I would use the Mobistudy app in the future to perform clinical tests such as the 6-minute walk test or the timed up and go test. |
|  |  | BIA2 | I would use the Mobistudy app in the future to share other data related to my physical health. |
| Usage Behaviour | wrist-worn device | UBW1 | How would you describe your use of the wrist-worn device? |
|  |  | UBW2 | What difficulties (if any) did you experience when using the wrist-worn device? |
|  | leg-worn device (only HBG) | UBL1 | How would you describe your use of the leg-worn device? |
|  |  | UBW2 | What difficulties (if any) did you experience when using the leg-worn device? |
|  | app | UBA1 | How would you describe your use of the Mobistudy app? |
|  |  | UBA2 | What difficulties (if any) did you experience when using the Mobistudy app? |

**Interviews after Day 60**

The semi-structured interviews will last around 30 minutes and be based on the mediators in the table below. The following questions provide a foundation for the interview:

**Table S4: Interviews after day 60**

| Mediator | Device | Measurement items |
| --- | --- | --- |
| Match with expectations | wrist-worn device | How was your experience of using the wrist-worn device? |
|  | leg-worn device (only HBG) | How was your experience of using the leg-worn device? |
|  | app | How was your experience of using the Mobistudy app? |
| Engagement | wrist-worn device | How has the wrist-worn device affected your interest in physical activity?  How has your attitude towards using a wrist-worn device changed? |
|  | leg-worn device (only HBG) | How has the leg-worn device affected your interest in physical activity?  How has your attitude towards using a leg-worn device changed? |
|  | app | How has the Mobistudy app affected your interest in physical activity?  How has your attitude towards a health app changed? |
| Coping strategies (only UK) | wrist-worn device | How has the wrist-worn device affected how you cope with your medical condition? |
|  | app | How has the Mobistudy app affected how you cope with your medical condition? |
| Perceived privacy | wrist-worn device | In terms of privacy, how did you feel using the wrist-worn device in your daily life? When did you feel more/less positive towards using it? |
|  | leg-worn device (only HBG) | In terms of privacy, how did you feel using the leg-worn device in your daily life? When did you feel more/less positive towards using it? |
|  | app | In terms of privacy, how did you feel using the Mobistudy app in your daily life? When did you feel more/less positive towards using it? |
| Resistance to change | wrist-worn device | If you felt reluctant (or keen) to use the wrist-worn device, could you explain why? |
|  | leg-worn device (only HBG) | If you felt reluctant (or keen) to use the leg-worn device, could you explain why? |
|  | app | If you felt reluctant (or keen) to use the app, could you explain why? |
| Possible negative experience | wrist-worn device | If you had to highlight one negative aspect from your use of the wrist-worn device, how would you describe this one? |
|  | leg-worn device (only HBG) | If you had to highlight one negative aspect from your use of the leg-worn device, how would you describe this one? |
|  | app | If you had to highlight one negative aspect from your use of the Mobistudy app, how would you describe this one? |
| Possible positive experience | wrist-worn device | If you had to highlight one positive aspect from your use of the wrist-worn device, how would you describe this one? |
|  | leg-worn device (only HBG) | If you had to highlight one positive aspect from your use of the leg-worn device, how would you describe this one? |
|  | app | If you had to highlight one positive aspect from your use of the Mobistudy app, how would you describe this one? |
| General feedback | wrist-worn device | What would make using the wrist-worn device more meaningful to you? |
|  | leg-worn device (only HBG) | What would make using the leg-worn device more meaningful to you? |
|  | app | What would make using the Mobistudy app more meaningful to you? |
| Trust | all | Would you trust this technology if used to make medical decisions by your doctor, such as:  For when you need to see your doctor?  For changes in medication?  Why? |

**Experience-based co-design workshops after Day 60**

We are planning to run 2 experience-based co-design workshops in both sites, Oxford and Helsingborg: a first workshop with 5 to 10 participants, and an optional follow-up workshop. The workshops will focus on discussing strengths and limitations of the current technology, to elaborate design ideas for improving the technology and future research. They will be based on a participatory design format that applies co-design methods that have previously been used in research projects in the area of digital health such as brainstorming or sketching. This form of generative sessions complement traditional ways for collecting qualitative data such as interviews since they focus not only on explicit but also on tacit and latent knowledge.

In the following, the format of the workshop is outlined. The format of the first and follow-up workshops is similar, with the second event focusing on iterating over ideas produced in the first. It is important to emphasise that the duration and scope of the activities may need to be adapted on site to the potential needs of the participants, such as additional breaks due to fatigue.

**Before the workshop**

The participants that expressed their interest to participate will be contacted by the researchers and invited to the workshop which will take place at a facility that is within the range of their usual interaction with the community centre (Helsingborg) or the clinic (Oxford). The participants will also be informed about their rights (that they can cancel their participation at any time), which type of data will be collected during the workshop (notes and graphical materials) and how they are going to be processed (for the purpose of understanding the role of technology with their health). Contact will happen by phone or email.

**Introduction (around 10 minutes)**

A short introduction related to the objective and the scope of the workshop will be provided. The facilitator(s) will introduce themselves and describe the intended format of the session. The previously communicated information about the participants’ rights and the planned data collection and processing will be also reminded.

**Warm-up activity (around 5-10 minutes)**

A warm-up activity such as a social interaction icebreaker will be conducted to help the participants to set the mood for the workshop. The aim is to establish an open and trustworthy environment where the participants feel comfortable to express their opinion and to share ideas. Another objective is that participants start to get to know each other.

**Technology experience-related activity (around 25-35 minutes)**

The participants will share some of their experiences with the technology in smaller groups and document it through post-its, simple sketches or similar artefacts. The facilitators will help to moderate the room and to group or map some of the participants’ experiences.

**Break (around 20 minutes)**

**Activity focussed on ideation (around 35-45 minutes)**

The facilitators will apply ideation methods commonly used in co-design processes applied in digital health projects to elicit concepts of how to develop the technology further based on elements that the participants consider as relevant or helpful in relation to their previous experience and daily life. Sketching or collages might be used as a visual documentation and may be complemented by interactive formats such as roleplaying or bodystorming.

**Wrapping up and communicating next steps (around 10-15 minutes)**

The facilitators will close the workshop and provide information about the next steps related to the collected data and discuss ideas for future research. The participants will be asked if they want to participate in a follow-up session (only for the first workshop).
