# Supplementary material for: Linking everyday physical activity and capacity tests using wearable and mobile technologies in older adults and cardiac cohorts: protocol for a pilot observational study
Source: BMJ Open. 2026 Feb 12;16(2):e112539. doi: 10.1136/bmjopen-2025-112539 (PMC12911791; doi:10.1136/bmjopen-2025-112539)
Supplement: Supplementary file 2 [file bmjopen-2025-112539.reviewer_comments.pdf]

PEER REVIEW HISTORY

BMJ Open publishes all reviews undertaken for accepted manuscripts. Reviewers are asked to complete a checklist review form and are provided with free text boxes to elaborate on their assessment. These free text comments are reproduced below.

ARTICLE DETAILS

Title (Provisional)

Linking everyday physical activity and capacity tests using wearable and mobile technologies in older adults and cardiac cohorts: protocol for a pilot observational study

Authors

Caramaschi, Sara; Maus, Benjamin; Olsson, Carl Magnus; Smedberg, Daniel; Kristen, Helmuth; Whitehead, Millie; Orchard, Elizabeth; Salvi, Dario

---

VERSION 1 - REVIEW

**Reviewer**	**1**
**Name**	**Strudwick, Gillian**
**Affiliation**	**Centre for Addiction and Mental Health, Information Management Group**
**Date**	**08-Dec-2025**
**COI**	**None**

Thanks for the opportunity to review your paper. I have several suggestions for the authors to consider.

1) The title is not very descriptive of what the study is about, nor the methods. This could be revised. There is nothing about wearable devices mentioned in the title at all, nor the clinical area.

2) More background on the clinical use of the 6MWT globally would be helpful to understand the potential impact of this study, as well as the clinical areas that may benefit. Its very high level at the moment/not specific.

3) The authors may wish to look at the flow and organization of the introduction section such that it builds more smoothly to the RQs. It reads a bit disjointed currently.

4) In the introduction, I would like to know a bit more about reference 6 and 7 as they directly relate to the feasibility of this study.

5) I don't understand this statement. Perhaps it can be re-worded or clarified? "Despite the high potential, there is little understanding of how to interpret data from

wearables in terms of clinical significance (19)"

6) This section seems unnecessary: "The article is organized as follows: The Introduction above outlined the study's context, aims

and research questions. The Materials and Methods section details the participants, technology used, study design, and data collection, analysis and management approaches. The Limitations and Strengths section reflects on such considerations. Following, Ethical

Considerations are proposed and at last, the final section of Conclusions".

7) I am not sure that RQ5 can truly be answered in an observational study, but rather it could be explored for some preliminary evidence that could be used in a more focused study for this RQ.

8) For the technology section and the strengths highlights section, suggest not using bullet points. I would also eliminate the numbering in the study design section.

9) How were the various wearables selected?

10) Will there be any patient engagement/patients part of designing the study such that uptake of these is appropriate? In #8 is the research design section, it refers to this but I am not clear if this happens before or after implementation of the wearables.

11) Consider using a STROBE or related checklist to ensure all items have been reported in your observational study

12) Statistical analyses planned could be better described

VERSION 1 - AUTHOR RESPONSE

Response to Reviewer 1:

We thank Dr. Gillian Strudwick for the helpful feedback. We did our best to include it in this revised version of the document and believe that the added edits significantly improved the manuscript. Following, we present a line-by-line explanation of the changes made. The black text corresponds to the reviewer's comments, while dark blue text represents the authors' answers to the reviewer's comments.

Thanks for the opportunity to review your paper. I have several suggestions for the authors to consider.

1) The title is not very descriptive of what the study is about, nor the methods. This could be revised. There is nothing about wearable devices mentioned in the title at all, nor the clinical area.

Thanks to the reviewer for the comment. The title was edited to be more representative and informative of the study aim from “Synergy Digital Health: a pilot study protocol” to “Linking everyday physical activity and capacity tests using wearable and mobile technologies in older adults and cardiac cohorts: an observational pilot study protocol”.

2) More background on the clinical use of the 6MWT globally would be helpful to understand the potential impact of this study, as well as the clinical areas that may benefit. Its very high level at the moment/not specific.

Thanks to the reviewer for the comment. While improving the introduction's flow and readability, we added some clarifications regarding the use of physical tests and the clinical areas involved as well as better definition of the used terminology. In particular, we specified the alternatives approaches that exist to measure capacity in cardiac patients (Oliveira MF, Zanussi G, Sprovieri B, Lobo DML, Mastrocolla LE, Umeda IIK, et al. Alternatives to Aerobic Exercise Prescription in Patients with Chronic Heart Failure. Arq Bras Cardiol. 2016 Feb;106(2):97–104.); we improved the terminology used across the manuscript with terms such as **physical performance and physical capacity** *(Implemented ICF Update Proposals 2018 [Internet]. [cited 2025 Dec 18]. Available from: https://www.who.int/publications/m/item/implemented-icf-update-proposals-2018)* and **digital health technologies** *(Yeung AWK, Torkamani A, Butte AJ, Glicksberg BS, Schuller B, Rodriguez B, et al. The promise of digital healthcare technologies. Front Public Health. 2023 Sept 26;11:1196596; Health C for D and R. What is Digital Health? FDA [Internet]. 2024 Dec 5 [cited 2026 Jan 2]; Available from: https://www.fda.gov/medical-devices/digital-health-center-excellence/what-digital-health)*

3) The authors may wish to look at the flow and organisation of the introduction section so that it builds more smoothly to the RQs. It reads a bit disjointed currently.

We understand and agree with the reviewer’s perspective. The introduction has been restructured to improve flow and readability. In particular, we stress the clinical relevance of the physical tests in the two types of cohorts, we better define terminology and separate logically the paragraphs.

4) In the introduction, I would like to know a bit more about reference 6 and 7 as they directly relate to the feasibility of this study.

A clearer explanation was added regarding references 6 and 7 as follows: “This is especially crucial for patients with or at risk of limited mobility who significantly struggle to perform the gold standard maximal test, the cardiopulmonary exercise test (CPET) (6). In these cases, the submaximal 6MWT and TUG are suitable for administration at follow-up appointments to objectively assess changes in symptoms, physical function

and capacity and overall health status. These tools are commonly used in clinical studies, for example, with elderly people, where the 6MWT was found to indicate overall health (7), and in cardiac patients, such as those who underwent the Fontan procedure (8).

5) I don't understand this statement. Perhaps it can be re-worded or clarified? "Despite the high potential, there is little understanding of how to interpret data from wearables in terms of clinical significance (19)"

The statement highlights one of the outcomes of the referenced systematic literature review. It is clarified as follows: “Despite the high potential of DHTs, there is a lack of consensus and standard procedures for processing and using continuous data collected from these devices from a clinical perspective (23).”

6) This section seems unnecessary: "The article is organised as follows: The Introduction above outlined the study's context, aims and research questions. The Materials and Methods section details the participants, technology used, study design, and data collection, analysis and management approaches. The Limitations and Strengths section reflects on such considerations. Following, Ethical Considerations are proposed and at last, the final section of Conclusions".

We agree with the reviewer and editor. The paragraph was removed.

7) I am not sure that RQ5 can truly be answered in an observational study, but rather it could be explored for some preliminary evidence that could be used in a more focused study for this RQ.

We thank the reviewer for this reflection on RQ5. We rephrased the RQ5 from: “Is the Timed Up and Go test a valid alternative to the 6MWT?” to “Are the results of the Timed Up and Go test associated to the results of the 6MWT?” In this way, we lower the aim to simple association rather than a full validation of the test.

8) For the technology section and the strengths highlights section, suggest not using bullet points. I would also eliminate the numbering in the study design section.

We edited the technology section, removing bullet points and improving the reading flow. At the same time, the strengths and limitations section was merged with the conclusion paragraphs, as suggested by the journal editors. We believe that the numbering within the study design section facilitates the reading flow and understanding of different steps of the protocol, and therefore, we decided not to edit this aspect of the manuscript.

9) How were the various wearables selected?

Multiple reasons lead us to have this technology selection. Wearables selection includes both medical-grade reference devices and consumer devices such as smartphones and wrist-worn devices. The G-Walk was selected as a reference device during the performance of the physical tests, given its already validated use in similar literature and due to the obtained medical device certification.
Given that users are asked to use devices often (or continuously for the wrist-worn), there is a need for comfort and acceptability of the technology, making personal smartphones and wrist-worn devices suitable. The MiBand was selected because of its affordability and because of having been used and validated in a long list of studies, for example:

- Bent, Brinnae, et al. "Investigating sources of inaccuracy in wearable optical heart rate sensors." NPJ digital medicine 3.1 (2020): 18.
- Chow, Hsueh-Wen, and Chao-Ching Yang. "Accuracy of optical heart rate sensing technology in wearable fitness trackers for young and older adults: validation and comparison study." JMIR mHealth and uHealth 8.4 (2020): e14707.
- Degroote, Laurent, et al. "Low-cost consumer-based trackers to measure physical activity and sleep duration among adults in free-living conditions: validation study." JMIR mHealth and uHealth 8.5 (2020): e16674.
- Xie, Junqing, et al. "Evaluating the validity of current mainstream wearable devices in fitness tracking under various physical activities: comparative study." JMIR mHealth and uHealth 6.4 (2018): e9754.

Moreover, the leg-worn device, Snubblometer, is developed by Infonomy, a partner in this research.

A clarification regarding technology selection is provided in the manuscript as follows: "The so-called DHTs used in this study are participants' smartphones, the Xiaomi Mi Band 3[1], and the Snubblometer[2]. In addition, during the first baseline visit, the medical-grade device G-Walk[3] is used as an additional reference to the standard clinician assessment and to the reference collected via a trundle wheel. This device selection includes a medical-grade device for use in clinic and consumer off-the-shelf devices for home monitoring. This allows the alignment of robust and validated devices, inexpensive consumer devices, and reference collected through standard ways from researchers and clinicians (using a stopwatch for the TUG or measuring the covered walked distance considering a known path for the 6MWT)."

10) Will there be any patient engagement/patients part of designing the study such that uptake of these is appropriate? In #8 is the research design section, it refers to this but I am not clear if this happens before or after implementation of the wearables.

We specify that patients and potential participants were included in the design of the study and also, we specify how we intend to disseminate the research study results with the involved participants.

This was added to the manuscript in the Participant and Public Involvement section as follows: "For the UK cohort, patients with a Fontan circulation have been involved in

designing the study. For the Swedish cohort, people from the local town administration, in direct contact with the community of older adults, were involved in the study design. At the end of the study, each participant receives a summary report of the data collected during the monitoring period. The summary can be discussed with healthcare professionals who are in contact with the participant."

11) Consider using a STROBE or related checklist to ensure all items have been reported in your observational study

We agree with the reviewer that using a defined checklist is relevant when determining the observational study protocol. We did consider the STROBE checklist; we referenced it in the Ethics and dissemination section of the manuscript and added the Word document to the manuscript's attachments on the submission portal.

12) Statistical analyses planned could be better described

The statistical analysis section of the manuscript was significantly improved in the paragraph "Data collection and analysis" of the "Methods and analysis" section. In particular, we 1) included an initial subparagraph related to the assessment of data quality and reliability of data sources, 2) included considerations about the minimal detectable changes in the physical tests and how they usually change (or do not) across time and multiple assessments, 3) added details and relevant literature to the analysis that we will adopt for RQ1 and RQ2 in terms of statistical methods and estimation methods.

VERSION 2 - REVIEW

Reviewer 1

Name Strudwick, Gillian

Affiliation Centre for Addiction and Mental Health, Information Management Group

Date 28-Jan-2026

COI

They took the suggestions seriously and did a thorough job editing the manuscript.
